# Effect of TiO_2_ Nanoparticle Addition on the Tribological Properties of CNT Coatings

**DOI:** 10.3390/ma18225092

**Published:** 2025-11-09

**Authors:** Sung-Jun Lee, Dae-Gyun Nam, Chang-Lae Kim

**Affiliations:** Department of Mechanical Engineering, Chosun University, Gwangju 61452, Republic of Koreadaegyun8086@hanmail.net (D.-G.N.)

**Keywords:** carbon nanotubes, titanium dioxide nanoparticles, tribological properties, spin coating, nanocomposite

## Abstract

Carbon nanotube (CNT) coatings show excellent tribological properties but face challenges in dispersion and industrial application. This study investigated TiO_2_ nanoparticle incorporation effects on CNT coating tribological performance. CNT/TiO_2_ composite coatings with varying TiO_2_ content (0.5–2.0 wt.%) were fabricated on SUS 304 substrates via spin coating. Surface morphology, roughness, wettability, and tribological properties were characterized using confocal microscopy, SEM, Raman spectroscopy, and reciprocating friction tests. Results showed that low TiO_2_ concentrations (0.5–0.7 wt.%) achieved optimal performance. The C3-Ti0.5 specimen maintained substrate-level smoothness (Ra = 0.09 μm) while preserving coating integrity. Raman analysis confirmed structural preservation of CNTs (ID/IG ≈ 1.0) across all formulations. Tribologically, C3-Ti0.5 exhibited a friction coefficient of 0.099, approaching pure CNT coating performance (0.090), with a wear rate of 9.00 × 10^−7^ mm^3^/N·mm. Higher TiO_2_ concentrations progressively degraded performance, with C3-Ti2 showing increased friction (0.263) and wear rate (2.87 × 10^−6^ mm^3^/N·mm). The 0.5–0.7 wt.% TiO_2_ range represents optimal composition for applications requiring both smooth surface finish and superior tribological performance, particularly for precision mechanical components where surface quality and friction control are equally critical.

## 1. Introduction

Currently, friction and wear phenomena in mechanical components are major causes of energy loss and equipment failure in industries, resulting in substantial economic losses [1]. Recognizing the severity of these problems, various nanoparticle-based coating technologies are being actively researched to improve low-friction and low-wear performance. In particular, light metals such as titanium, aluminum, and magnesium are widely utilized in the aerospace, automotive, marine, and defense industries because of their low density and high specific strength characteristics [2]. However, these materials are vulnerable to friction and wear under mechanical contact conditions, such as sliding, rolling, and rotating motions, which significantly reduce the component life and efficiency. Therefore, it is essential to improve the tribological properties of contact interfaces to improve the performance and durability of mechanical components [3].

To achieve these objectives, carbon-based nanomaterials with excellent physicochemical properties, including graphite, carbon nanotubes (CNTs), fullerenes, and graphene, have been extensively studied [4,5]. Among these, CNTs have received significant attention as next-generation solid lubricants owing to their outstanding mechanical strength and stiffness, excellent electrical and thermal conductivity, and exceptional lubricating properties derived from their unique hollow structure. However, CNTs face challenges in achieving uniform dispersion within matrices due to strong van der Waals forces that cause agglomeration, and their high manufacturing costs limit their industrial applications [6]. Recent investigations have explored various approaches to improve CNT-based tribological coatings. Plasma-sprayed TiO_2_/CNT coatings have demonstrated significant wear volume reduction through carbothermic interfacial bonding, where Ti_n_O_2n−1_ phases form at the CNT-matrix interface, facilitating effective stress transfer mechanisms [7]. The tribological performance of these coatings was attributed to multiple frictional effects, including tribo-reorientation, tribo-protruding, tribo-film formation, and controlled tribo-degradation processes.

Titanium dioxide (TiO_2_) is a representative metal oxide nanomaterial utilized in various industrial fields owing to its excellent mechanical strength, chemical stability, and photocatalytic properties [8]. When CNTs and TiO_2_ nanoparticles are combined, the strong bonding force of TiO_2_ improves the dispersion of CNTs, and the synergistic effect between the two materials can significantly enhance their mechanical properties and lifespan. Additionally, the interfacial structure formed by the TiO_2_ nanoparticles increases the stress distribution efficiency within the CNT matrix, improving the friction and wear characteristics while enhancing the surface activity [9,10]. However, excessive addition of TiO_2_ nanoparticles can cause agglomeration, reducing interfacial adhesion and consequently decreasing the mechanical properties of the composite [11,12].

Various techniques have been developed to fabricate CNT/TiO_2_ nanocomposite coating surfaces, including dip coating, spin coating, and electrospinning [13,14]. Among these, spin-coating technology is highly promising for industrial applications because of its simple process that can form uniform coating films over large areas, along with high reproducibility and cost-effectiveness [15,16].

Alternative coating architectures have been developed to combine the advantages of different nanomaterials. Bilayer coating systems incorporating anodized TiO_2_ nanotube arrays with subsequently deposited carbon nanostructures have shown promise for biomedical applications [17]. These hierarchical structures demonstrate that the morphology of TiO_2_ layers significantly influences the subsequent carbon deposition, resulting in diverse nanostructures ranging from nanoflakes to nano-rods depending on the substrate preparation.

Comparative tribological studies under reciprocating contact conditions have revealed the importance of testing methodology in evaluating coating performance. Investigations comparing different materials under ball-on-plate configurations with controlled environmental conditions have established baseline performance metrics for various coating systems [18]. These studies emphasize the critical role of contact geometry and lubrication conditions in determining wear mechanisms and coating durability.

In this study, CNT/TiO_2_ nanocomposite coatings were synthesized through a spin-coating process to improve the friction and wear performance of mechanical components, and the effects of TiO_2_ nanoparticle content on tribological properties were investigated. The surface morphology, roughness, wettability, and friction and wear resistance were evaluated to determine the optimal TiO_2_ content, with the aim of developing nanocomposite coatings with enhanced mechanical properties. The results of this study are expected to serve as important foundational data for developing surface treatment technologies to improve the performance and extend the lifespan of mechanical components.

## 2. Materials and Methods

### 2.1. Materials

In this study, multi-walled carbon nanotubes (MWCNTs; Wonil Co., Ltd., Yangsan, Republic of Korea) were used as solid lubricants. TiO_2_ nanoparticles (20 nm average particle size; Sigma-Aldrich, St. Louis, MO, USA) were used as the additives. Isopropyl alcohol (Duksan Co., Ltd., Ansan, Republic of Korea) and ethanol (Duksan Co., Ltd., Ansan, Republic of Korea) were used as the solvents. Stainless steel (SUS) 304 stainless-steel substrates (20 mm × 20 mm × 1 mm) were used as the substrate for the coating.

### 2.2. Preparation of Coating Solutions

The CNT solution was prepared by dispersing MWCNTs in isopropyl alcohol at a concentration of 3 wt.%. TiO_2_ solutions were prepared by dispersing TiO_2_ nanoparticles in ethanol at various concentrations of 0.5, 0.7, 0.9, 1.2, 1.5, and 2.0 wt.%. The CNT and TiO_2_ solutions were mixed at a 1:1 volume ratio, followed by mechanical stirring at 300 rpm for 30 min. Subsequently, additional dispersion treatment was performed for 60 min using an ultrasonic processor to ensure uniform mixing and stable dispersion between CNT and TiO_2_ nanoparticles.

### 2.3. Coating Process

**Figure 1:**
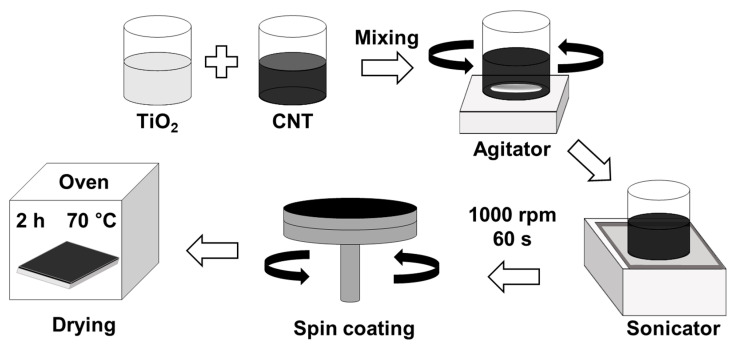
Schematic illustration of the fabrication process for CNT/TiO_2_ nanocomposite coatings via the spin-coating method.

The coating process is illustrated in Figure 1. SUS 304 substrates were ultrasonically cleaned for 10 min each in acetone, ethanol, and distilled water sequentially to remove surface contaminants. After dropping 0.5 mL of the prepared CNT/TiO_2_ composite solution onto the cleaned substrate, spin-coating was performed at a rotation speed of 1000 rpm for 60 s. The coated specimens were dried in a hot air drying chamber at 70 °C for 2 h to completely remove the solvent and enhance the adhesion of the coating layer.

The fabricated specimens were designated as follows: the uncoated substrate was designated as Bare, and the specimen coated with pure CNT only was designated as C3. The composite coating specimens with added TiO_2_ nanoparticles were designated as C3-Ti0.5, C3-Ti0.7, C3-Ti0.9, C3-Ti1.2, C3-Ti1.5, and C3-Ti2 according to the TiO_2_ content.

### 2.4. Characterization

Raman spectroscopy was performed using a 532 nm laser source with a 100× objective lens. A neutral density filter (5%) was used to prevent sample damage. Spectra were collected in the range of 500–3500 cm^−1^ with an acquisition time of 1 s and 30 accumulations to improve the signal-to-noise ratio. The D band (1350 cm^−1^), G band (1580 cm^−1^), and 2D band (2700 cm^−1^) were analyzed, and the ID/IG ratio was calculated to evaluate the structural quality of the CNT coatings.

The surface morphologies of the fabricated coatings were observed using a confocal microscope (VK-X200, Keyence Co., Ltd., Osaka, Japan) and scanning electron microscopy (SEM; SU-8600, Hitachi, Tokyo, Japan). To evaluate the wettability of the coating solutions, 5 μL of the coating solution was dropped onto the bare substrate using a micropipette, and the contact angles were measured using digital microscopy. Surface roughness and coating thickness were evaluated using a surface roughness tester (SV-2100M4, Mitutoyo Korea Corporation, Gunpo, Republic of Korea) under conditions of 2 mm evaluation length and 0.2 mm/s measurement speed, with measurements taken five times per specimen to calculate average values.

The friction and wear characteristics were evaluated using a reciprocating friction tester (RFW 160, Neoplus Co., Ltd., Daejeon, Republic of Korea) in accordance with ASTM G133 [19,20]. The reciprocating test configuration was selected to simulate micro-scale tribological conditions encountered in precision mechanical systems such as MEMS devices, micro-actuators, and precision bearings, where localized contact and small-amplitude oscillations are prevalent. A stainless steel 304 (SUS 304) ball with a diameter of 1 mm and surface roughness of Ra < 0.1 μm was used as the counterpart for the tests. Tests were performed for 2000 cycles under conditions of 2 mm stroke and 4 mm/s sliding speed. The vertical load was set at 50 mN, and all tests were conducted in an atmospheric environment at room temperature (25 °C) and 50% relative humidity (RH). Each test condition was repeated at least three times to ensure statistical reliability, and the reported friction coefficient curves and wear measurements represent the average values from these repetitions. The friction coefficients were measured and recorded in real time, and the morpAhology of the wear tracks and wear mechanisms was analyzed using confocal microscopy and SEM.

## 3. Results and Discussion

### 3.1. Surface Structural and Morphology Analysis

**Figure 2:**
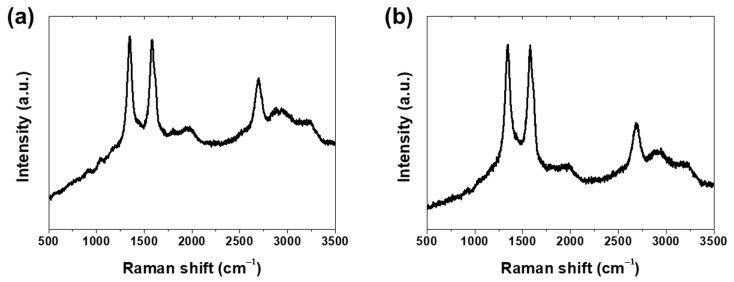
Raman spectra of (**a**) pure CNT coating (C3) and (**b**) CNT/TiO_2_ composite coating (C3-Ti2).

Raman spectroscopy was performed to investigate the structural characteristics of the coatings, as shown in Figure 2. Both the pure CNT coating (C3) in Figure 2a and the CNT/TiO_2_ composite coating (C3-Ti2) in Figure 2b exhibited characteristic peaks at 1350 cm^−1^ (D band), 1580 cm^−1^ (G band), and 2700 cm^−1^ (2D band) [21,22]. The intensity ratio of the D band to the G band (ID/IG) was approximately 1.0 for both specimens, indicating a similar degree of structural disorder. The comparable ID/IG ratios between C3 and C3-Ti2 suggest that the incorporation of TiO_2_ nanoparticles did not introduce significant structural defects to the CNT network, maintaining the structural integrity of the carbon nanotubes in the composite coating.

**Figure 3:**
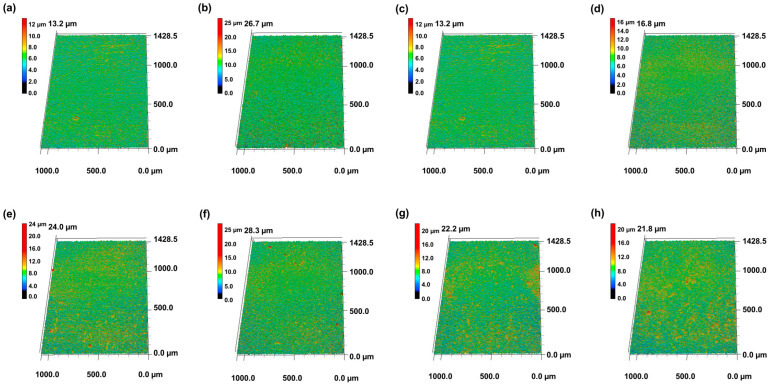
Confocal microscopy images of CNT/TiO_2_ nanocomposite coating surfaces with different TiO_2_ contents: (**a**) bare, (**b**) C3, (**c**) C3-Ti0.5, (**d**) C3-Ti0.7, (**e**) C3-Ti0.9, (**f**) C3-Ti1.2, (**g**) C3-Ti1.5, and (**h**) C3-Ti2.

**Figure 4:**
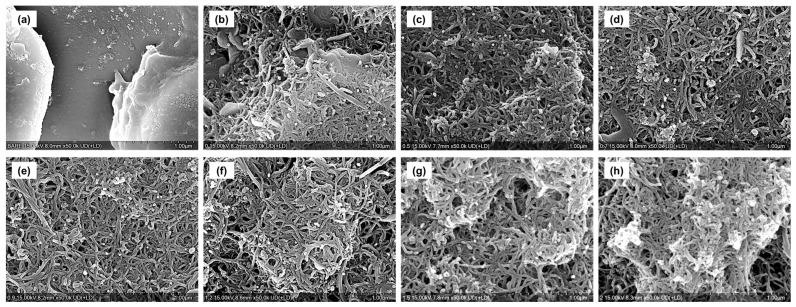
SEM images of coating surfaces for (**a**) bare, (**b**) C3, (**c**) C3-Ti0.5, (**d**) C3-Ti0.7, (**e**) C3-Ti0.9, (**f**) C3-Ti1.2, (**g**) C3-Ti1.5, and (**h**) C3-Ti2.

The surface morphologies of the CNT/TiO_2_ nanocomposite coatings fabricated via spin coating were observed using confocal microscopy and SEM, and the results are shown in Figure 3 and Figure 4. For the pure CNT coating (C3), a network structure formed by intertwined CNTs was observed, exhibiting a fine porous structure that was relatively uniformly dispersed by the centrifugal force of spin coating. This porous network structure is an inherent characteristic of CNTs and is expected to contribute to improved lubrication properties.

In the case of composite coatings with added TiO_2_ nanoparticles, distinct changes in the surface morphology were observed as the TiO_2_ content increased. In the C3-Ti0.5 specimen, TiO_2_ nanoparticles were uniformly dispersed within the CNT network structure, and the CNT agglomeration was significantly reduced. This is interpreted as the result of a small amount of TiO_2_ nanoparticles weakening the van der Waals forces between the CNTs, thereby improving dispersibility. In the C3-Ti0.7 and C3-Ti0.9 specimens, aggregates of several micrometers in size began to form with increasing TiO_2_ content, which was attributed to the adsorption of TiO_2_ nanoparticles onto the CNT surfaces and the formation of localized clusters [23]. In specimens with higher TiO_2_ content (C3-Ti1.2, C3-Ti1.5, and C3-Ti2), small granular microstructures with rough and uneven textures were observed.

**Figure 5:**
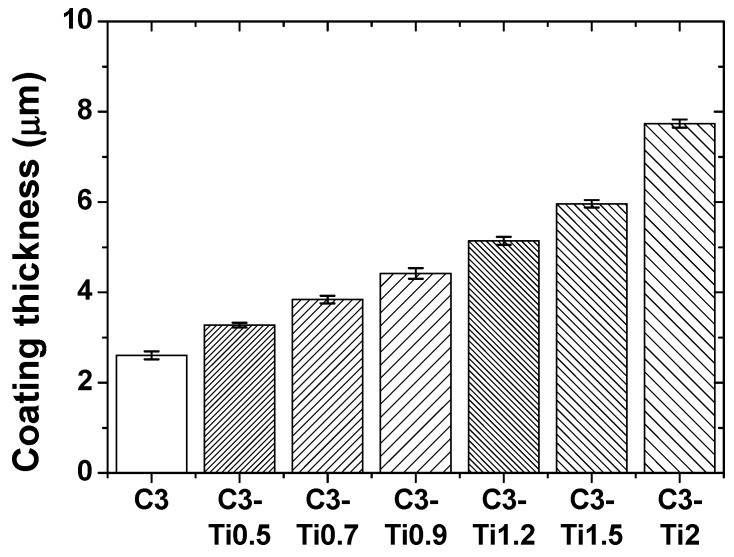
Coating thickness of CNT/TiO_2_ nanocomposite coatings with varying TiO_2_ content.

Coating thickness measurements revealed variation with TiO_2_ content (Figure 5). The pure CNT coating (C3) exhibited a thickness of 2.6 μm, while incorporation of TiO_2_ nanoparticles progressively increased the coating thickness: 3.27 μm for C3-Ti0.5, 3.84 μm for C3-Ti0.7, 4.42 μm for C3-Ti0.9, 5.14 μm for C3-Ti1.2, 5.96 μm for C3-Ti1.5, and 7.74 μm for C3-Ti2. This thickness increase corresponds to the additional volume contributed by TiO_2_ nanoparticles and their influence on coating deposition during spin coating. Despite the greater thickness of high TiO_2_ content specimens, their inferior tribological performance indicates that coating composition and microstructural integrity are more critical factors than thickness alone.

### 3.2. Surface Roughness Analysis

**Figure 6:**
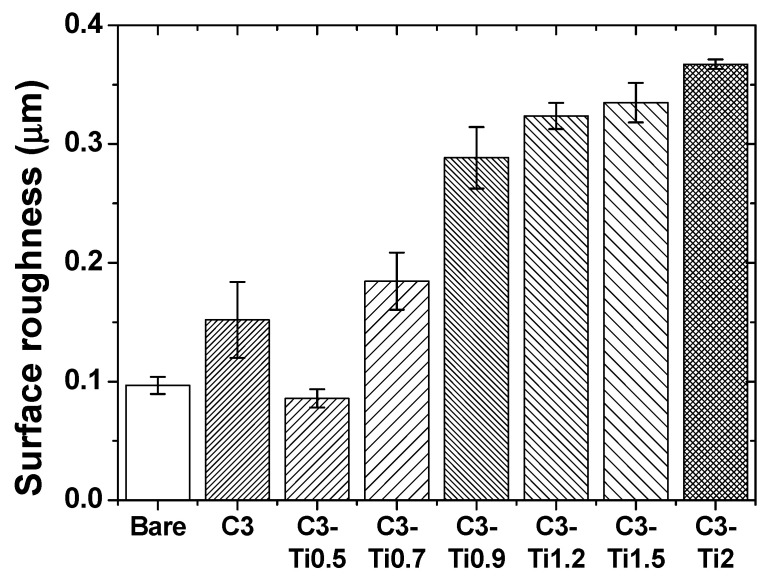
Surface roughness (Ra) values of CNT/TiO_2_ nanocomposite coatings as a function of the TiO_2_ content.

The surface roughness measurement results of the CNT/TiO_2_ nanocomposite coatings obtained via confocal microscopy are shown in Figure 6. The bare specimen exhibited a surface roughness (arithmetic average roughness, Ra) value of 0.09 μm, whereas the pure CNT coating (C3) exhibited an increased roughness of 0.15 μm. This indicates that the CNT coating moderately increased the substrate surface roughness.

For composite coatings with added TiO_2_, the surface roughness exhibited characteristic variation patterns depending on the content. In the C3-Ti0.5 specimen, the surface roughness remained at 0.09 μm, identical to that of the bare specimen, indicating that a small amount of TiO_2_ nanoparticles effectively filled the pores in the CNT network while maintaining a smooth surface [24]. This phenomenon is attributed to the selective adsorption of TiO_2_ nanoparticles onto defect sites in the CNT structure, contributing to surface planarization.

In the C3-Ti0.7 and C3-Ti0.9 specimens, the surface roughness increased to 0.18 and 0.29 μm, respectively. This can be attributed to the structural changes in the coating morphology with increasing TiO_2_ content, which is consistent with confocal microscopy observations. The C3-Ti0.9 specimen exhibited more than triple the roughness of the bare specimen, suggesting that TiO_2_ nanoparticles in this range modified the surface topology, forming localized protrusions that significantly increased the surface roughness.

The surface roughness continued to increase with increasing TiO_2_ content, with the C3-Ti1.2 specimen showing 0.32 μm and the C3-Ti1.5 specimen recording 0.33 μm. Notably, the C3-Ti2 specimen exhibited the highest roughness value of 0.37 μm, representing more than a four-fold increase compared with the bare specimen. This progressive increase in roughness suggests that a higher TiO_2_ content leads to more pronounced surface features and an increased surface texture. The monotonic increase in roughness with TiO_2_ content beyond C3-Ti0.5 indicates that the addition of nanoparticles continuously modified the surface morphology, resulting in progressively rougher surfaces at higher concentrations.

### 3.3. Wettability Analysis

**Figure 7:**
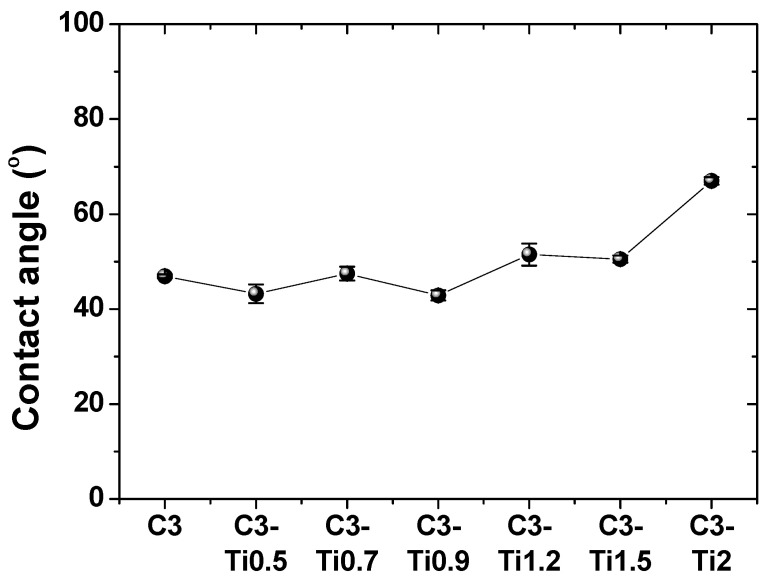
Contact angle measurements of CNT/TiO_2_ nanocomposite coating solutions on SUS 304 stainless steel substrate with varying TiO_2_ contents.

The wettability of the coating solutions is an important factor affecting the interaction with the substrates and coating quality. According to the contact angle measurement results shown in Figure 7, the contact angle of the pure CNT solution (C3) was 46.9°. This is attributed to the wettability and cohesive forces of the CNTs, indicating moderate wettability.

For composite coating solutions with added TiO_2_, characteristic contact angle variation patterns were observed depending on the content. The contact angle of the C3-Ti0.5 solution decreased to 43.2°, which resulted from the hydrophilic TiO_2_ nanoparticles partially mitigating the hydrophobicity of the CNTs. It increased to 47.4° at C3-Ti0.7, and then showed the lowest value of 42.8° at C3-Ti0.9. These changes are attributed to the dispersion state of the TiO_2_ nanoparticles and their degree of interaction with CNTs, where C3-Ti0.9 exhibits the best wettability owing to the optimal interaction between the two components.

In the C3-Ti1.2 solution, the contact angle sharply increased to 51.4° and was maintained at 50.5° for C3-Ti1.5. This was attributed to the agglomeration of nanoparticles when the TiO_2_ content exceeded a critical point, thereby changing the surface energy. The C3-Ti2 solution exhibited the highest contact angle of 67°, which is attributed to the severe agglomeration phenomena due to excessive TiO_2_, which increased the surface tension of the solution and significantly deteriorated wettability [25].

The progressive increase in contact angle with TiO_2_ content indicates increased solution viscosity due to nanoparticle incorporation. This viscosity change directly influences the spin coating process, where higher viscosity solutions resist centrifugal spreading, resulting in thicker coatings. The correlation between contact angles and measured coating thicknesses confirms this relationship: the C3 specimen with a 46.9° contact angle produced a 2.6 μm coating, while the C3-Ti2 specimen with a 67° contact angle yielded a 7.74 μm coating.

The intermediate wettability of C3-Ti0.5 and C3-Ti0.9 specimens represents a favorable balance between solution flowability and material deposition. These solutions spread sufficiently during spin coating to form uniform films while retaining adequate material to produce protective coatings. In contrast, the poor wettability of high TiO_2_ content solutions (C3-Ti1.5 and C3-Ti2) not only produced thicker coatings but also likely contributed to non-uniform material distribution, explaining the increased surface roughness observed in these specimens. These findings demonstrate that solution wettability serves as a predictive parameter for coating quality and thickness in spin coating processes.

### 3.4. Friction Characteristics Analysis

**Figure 8:**
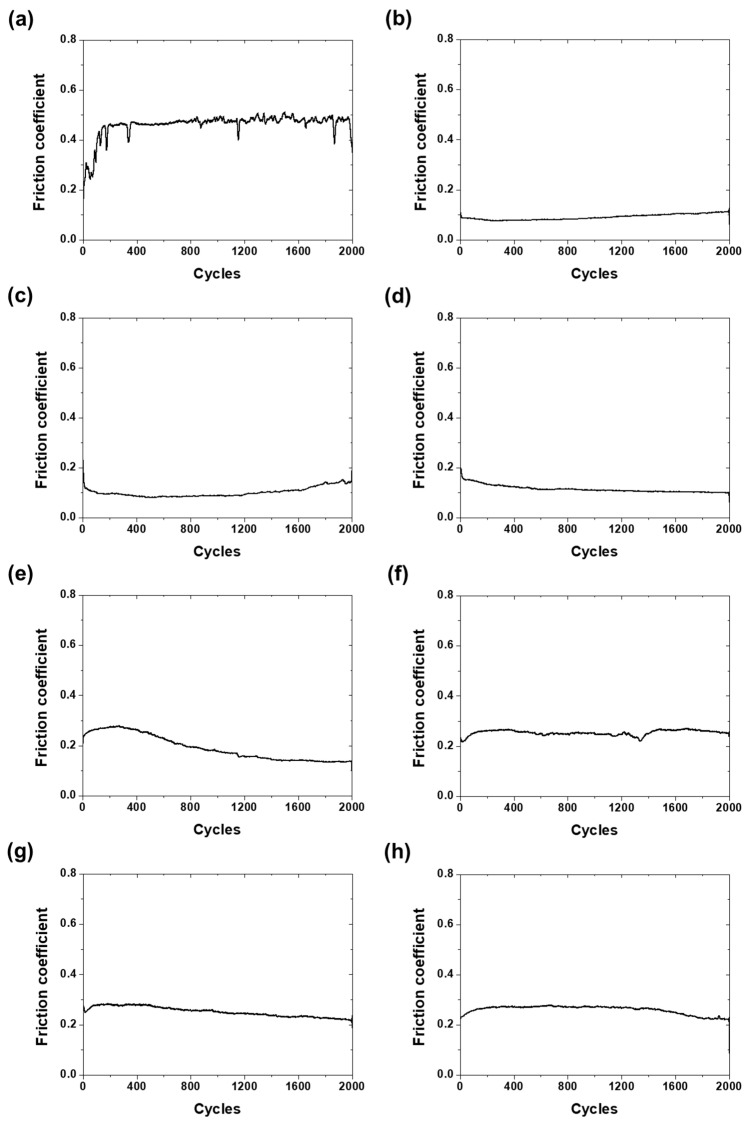
Friction coefficient as a function of sliding cycles for CNT/TiO_2_ nanocomposite coatings with different TiO_2_ contents: (**a**) bare, (**b**) C3, (**c**) C3-Ti0.5, (**d**) C3-Ti0.7, (**e**) C3-Ti0.9, (**f**) C3-Ti1.2, (**g**) C3-Ti1.5, and (**h**) C3-Ti2.

The results of the reciprocating friction tests performed to evaluate the friction characteristics of the CNT/TiO_2_ nanocomposite coatings are shown in Figure 8. The bare specimen started with an initial friction coefficient of approximately 0.3, which rapidly increased to above 0.45 within 200 cycles, and after experiencing slight fluctuations, finally reached 0.4. This represents the typical friction behavior in metal-to-metal contact, caused by the destruction of the initial surface oxide film and direct metal-to-metal contact.

The pure CNT coating (C3) maintained a low friction coefficient below 0.1 until 1200 cycles, after which it gradually increased to above 0.1. This demonstrates that the excellent solid lubricating properties of CNTs were maintained for an extended period owing to the layered structure and network form of CNTs, which acted as an effective lubricant film [26].

The friction behavior of the composite coatings with added TiO_2_ exhibited distinct differences depending on the content. The friction coefficient of the C3-Ti0.5 specimen sharply decreased from an initial value of 0.2 to 0.1, was maintained until 1200 cycles, and then gradually increased to 0.15. This suggests that a small amount of TiO_2_ formed an effective tribofilm with CNTs during the initial run-in process.

The C3-Ti0.7 specimen decreased from an initial 0.2 to 0.17 within 20 cycles, and then showed a continuously decreasing trend to finally reach the 0.1 level. This continuously decreasing tendency indicates that TiO_2_ nanoparticles gradually formed a stable lubricating film together with CNTs during the friction process.

The C3-Ti0.9 specimen started above the initial 0.2, increased to 0.3 by 200 cycles, and then sharply decreased to 0.2 by 1000 cycles. Subsequently, it showed a gradually decreasing trend, finally reaching 0.13. This complex behavior is interpreted as an initial friction increase due to TiO_2_ aggregates; however, during the continuous friction process, the aggregates decomposed and rearranged to form a stable lubricating film.

The C3-Ti1.2 specimen started at approximately 0.23, increased to 0.25 within the initial 100 cycles, experienced slight fluctuations, and finally maintained a value of 0.25. The C3-Ti1.5 and C3-Ti2 specimens showed an increasing tendency within the initial 50 cycles but subsequently decreased gradually, with both specimens finally reaching 0.21. The high friction coefficients of these high-content TiO_2_ specimens demonstrate that excessive TiO_2_ nanoparticles significantly impair the lubricating properties of CNTs.

**Figure 9:**
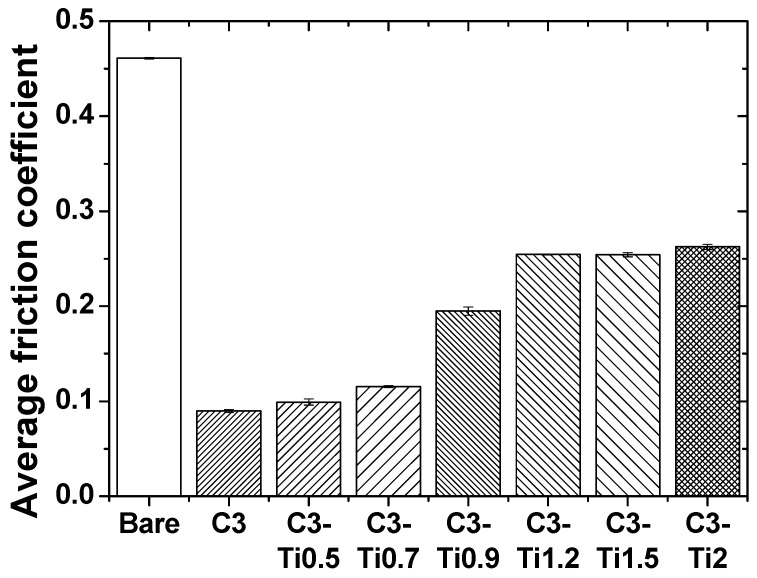
Average friction coefficient of CNT/TiO_2_ nanocomposite coatings with different TiO_2_ contents.

In the comparison of the average friction coefficients shown in Figure 9, the bare specimen exhibited the highest value of 0.461, whereas that of the pure CNT coating (C3) decreased sharply to 0.090, confirming the excellent lubricating properties of CNTs. Among the TiO_2_-added specimens, C3-Ti0.5 had the lowest average friction coefficient of 0.099, and as the TiO2 content increased, the values progressively increased: C3-Ti0.7 at 0.115, C3-Ti0.9 at 0.195, C3-Ti1.2 at 0.255, C3-Ti1.5 at 0.254, and C3-Ti2 at 0.263, showing a gradually increasing trend in the friction coefficient.

### 3.5. Wear Mechanism Analysis

**Figure 10:**
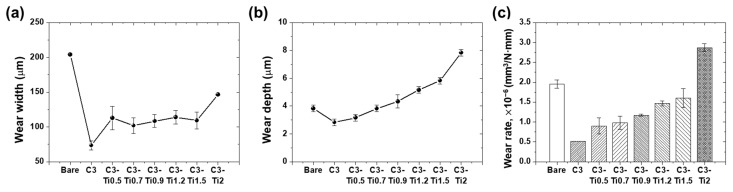
Wear characteristics of bare substrate and CNT/TiO_2_ composite coatings: (**a**) wear width, (**b**) wear depth, and (**c**) wear rate after 2000 cycles of reciprocating friction tests.

Wear measurements were performed to evaluate the wear resistance of the coatings. Figure 10 presents the wear width, wear depth, and wear rate for each specimen. The bare substrate exhibited the highest wear width of 204 μm and a wear depth of 3.83 μm. The pure CNT coating (C3) demonstrated superior wear resistance with the smallest wear width of 73.5 μm and a wear depth of 2.83 μm, resulting in the lowest wear rate of 5.19 × 10^−7^ mm^3^/N·mm.

Among the TiO_2_-containing specimens, C3-Ti0.7 showed the best wear resistance with a wear width of 102 μm and a wear rate of 9.81 × 10^−7^ mm^3^/N·mm, followed closely by C3-Ti0.9 (108.5 μm width, 1.17 × 10^−6^ mm^3^/N·mm wear rate). The wear depth progressively increased with TiO_2_ content, from 3.17 μm for C3-Ti0.5 to 7.83 μm for C3-Ti2. The C3-Ti2 specimen exhibited the poorest wear performance among the coated specimens, with a wear width of 146.5 μm and the highest wear rate of 2.87 × 10^−6^ mm^3^/N·mm, though still performing better than the bare substrate. These quantitative results confirm that low TiO_2_ concentrations (0.5–0.7 wt.%) maintain reasonable wear resistance, while higher concentrations progressively degrade the wear performance.

**Figure 11:**
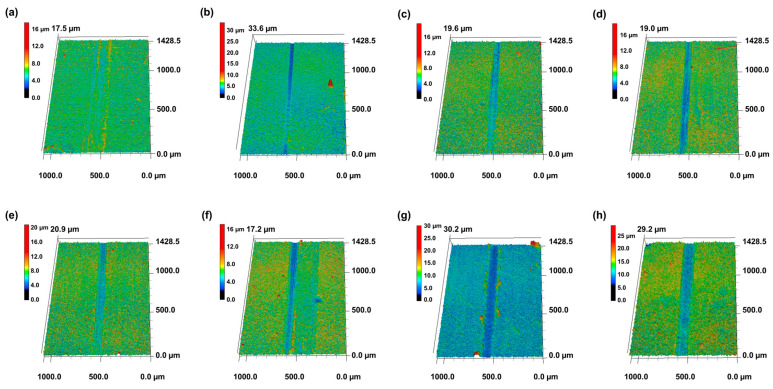
Confocal microscopy images of wear tracks after 2000 sliding cycles for CNT/TiO_2_ nanocomposite coatings with different TiO_2_ contents: (**a**) bare, (**b**) C3, (**c**) C3-Ti0.5, (**d**) C3-Ti0.7, (**e**) C3-Ti0.9, (**f**) C3-Ti1.2, (**g**) C3-Ti1.5, and (**h**) C3-Ti2.

**Figure 12:**
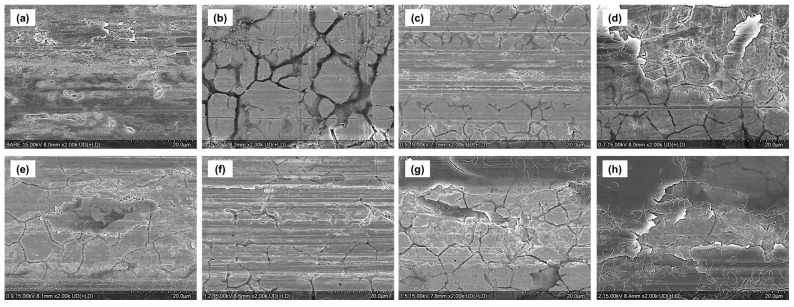
SEM images of wear tracks after 2000 cycles for (**a**) bare, (**b**) C3, (**c**) C3-Ti0.5, (**d**) C3-Ti0.7, (**e**) C3-Ti0.9, (**f**) C3-Ti1.2, (**g**) C3-Ti1.5, and (**h**) C3-Ti2.

The results of confocal microscopy and SEM observations of the wear tracks after friction testing are shown in Figure 11 and Figure 12. The bare specimen exhibited deep grooves and severe traces of abrasive wear, representing a serious wear phenomenon typical of metal-to-metal contact.

The pure CNT coating (C3) exhibited the narrowest and shallowest wear track among all specimens, demonstrating the best wear resistance. This is because the layered structure of CNTs acted as an effective solid lubricating film that prevented direct metal contact, and the network structure effectively distributed stress. Fine delamination and parallel scratch marks were observed along the edges of the wear track, indicating that micro plowing was the primary wear mechanism [27].

Among the TiO_2_-added specimens, C3-Ti0.5, C3-Ti0.7, and C3-Ti0.9 exhibited relatively narrow wear widths. In these specimens, fine cracks were observed around the wear tracks, which were caused by the tensile stress occurring during the sliding contact. In particular, the C3-Ti0.5 and C3-Ti0.7 specimens exhibited excellent wear resistance, second only to the CNT coating, suggesting that an appropriate amount of TiO_2_ exhibited synergistic effects with CNTs to enhance wear resistance.

In contrast, high-content specimens of C3-Ti1.2 and above showed wide wear tracks along with scratches on the substrate. This suggests that excessive TiO_2_ nanoparticles formed aggregates that acted as third-body abrasives. Extensive surface delamination and microcracks were observed along the wear tracks, and fragments of TiO_2_ aggregates were clearly identified within the wear tracks. These aggregates were interpreted to have destroyed the coating layer during the friction process and directly damaged the substrate.

The C3-Ti1.5 and C3-Ti2.0 specimens exhibited deep wear grooves along with traces of large-scale delamination and plastic deformation. This was attributed to the increased brittleness of the coating layer and weak interfacial bonding due to excessive TiO_2_, indicating that localized fatigue failure and adhesive wear occurred in combination. In particular, in areas where the substrate was exposed, serious metal-to-metal contact occurred, accelerating wear.

**Figure 13:**
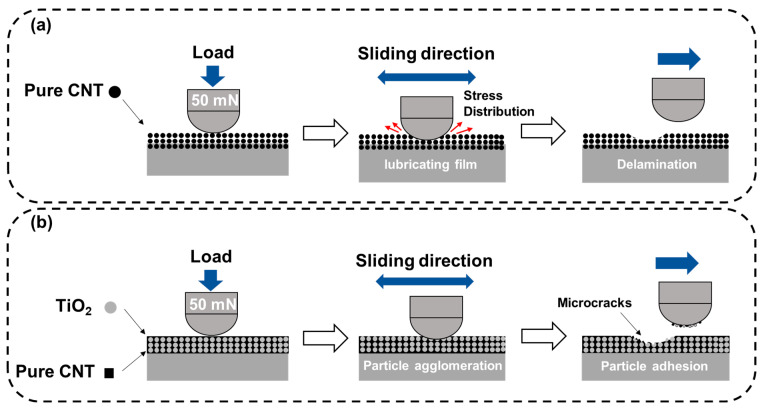
Schematic illustration of wear mechanisms: (**a**) pure CNT coating and (**b**) CNT/TiO_2_ composite coating.

Figure 13 illustrates the proposed wear mechanisms for the CNT and CNT/TiO_2_ coatings using schematic diagrams. Figure 13a depicts the wear mechanism of the pure CNT coating, where the CNT network structure bears the applied load and undergoes deformation during the wear process. The entangled CNT structure provides effective wear resistance through its inherent flexibility and load distribution capabilities. Figure 13b demonstrates the wear mechanism of the CNT/TiO_2_ composite coating, where TiO_2_ nanoparticles are incorporated within the CNT matrix. Despite the presence of TiO_2_ nanoparticles, the composite coating exhibited increased wear compared to the pure CNT coating. This may be attributed to the hard TiO_2_ particles acting as stress concentration sites or disrupting the flexible CNT network structure, thereby accelerating material removal during the wear process. Additionally, the TiO_2_ nanoparticles may have reduced the cohesive strength between the CNTs, resulting in easier detachment of the coating material under tribological stress.

## 4. Conclusions

This investigation into CNT/TiO_2_ nanocomposite coatings fabricated via spin coating revealed promising opportunities for tailored tribological surface treatments. The incorporation of TiO_2_ nanoparticles at controlled concentrations demonstrated the potential to achieve specific surface characteristics while maintaining favorable friction and wear properties.

A particularly significant finding was the achievement of ultra-smooth surfaces at low TiO_2_ concentrations, with the C3-Ti0.5 specimen matching the bare substrate roughness of 0.09 μm while retaining the protective coating layer. This combination of surface smoothness and coating integrity represents an advancement in precision engineering applications. The friction performance at these low TiO_2_ levels remained remarkably close to that of pure CNT coatings, with C3-Ti0.5 achieving 0.099 compared to 0.090 for pure CNT, demonstrating that carefully controlled nanoparticle addition need not compromise the fundamental lubricating properties of CNT networks.

The wear resistance evaluation further supported the viability of low-concentration TiO_2_ additions, with specimens containing 0.5–0.7 wt.% TiO_2_ exhibiting wear track morphologies similar to pure CNT coatings. The preservation of structural integrity, as evidenced by the consistent ID/IG ratios across all formulations, indicates that the fundamental architecture of the CNT network remains intact even with the incorporation of nanoparticles.

The current coating system relies on physical adhesion through solvent evaporation, which was deliberately selected to investigate the fundamental tribological properties of CNT/TiO_2_ composites without interference from binders. While this approach proved sufficient for laboratory-scale tribological evaluation, industrial implementation will require improved adhesion mechanisms. Future development should focus on three key areas: (1) substrate surface functionalization through plasma treatment or to increase coating-substrate interfacial bonding, (2) incorporation of silane coupling agents or polymer binders to achieve chemical bonding while minimizing effects on tribological properties, and (3) thermal or UV post-treatment processes to cross-link the coating structure and improve mechanical robustness.

The fundamental knowledge gained from this study regarding optimal TiO_2_ content and its effects on surface-tribological property relationships provides a critical foundation for developing industrially viable coating systems. The identified 0.5–0.7 wt.% TiO_2_ range represents the target composition for future formulations that incorporate adhesion-promoting strategies. Additionally, exploring hybrid reinforcement approaches and investigating the long-term environmental stability of these coatings under various service conditions will be essential for practical applications in precision mechanical components.

## Data Availability

The original contributions presented in this study are included in the article. Further inquiries can be directed to the corresponding author.
